# Improving water deficit tolerance of *Salvia officinalis* L. using putrescine

**DOI:** 10.1038/s41598-021-00656-1

**Published:** 2021-11-09

**Authors:** Maryam Mohammadi-Cheraghabadi, Seyed Ali Mohammad Modarres-Sanavy, Fatemeh Sefidkon, Sajad Rashidi-Monfared, Ali Mokhtassi-Bidgoli

**Affiliations:** 1 Department of Agronomy, Faculty of Agriculture, Tarbiat Modares University, PO Box 14115-336, Tehran, Iran; 2 Research Institute of Forests and Rangelands, Tehran, Iran; 3 Agricultural Biotechnology Department, Faculty of Agriculture, Tarbiat Modares University, Tehran, Iran

**Keywords:** Agricultural genetics, Plant sciences, Plant genetics, Plant physiology, Plant stress responses, Secondary metabolism

## Abstract

To study the effects of foliar application of putrescine (distilled water (0), 0.75, 1.5, and 2.25 mM) and water deficit stress (20%, 40%, 60%, and 80% available soil water depletion (ASWD)) on the physiological, biochemical, and molecular attributes of *Salvia officinalis* L., a factorial experiment was performed in a completely randomized design with three replications in the growth chamber. The results of Real*-*Time quantitative polymerase chain reaction (qRT-PCR) analysis showed that putrescine concentration, irrigation regime, and the two-way interaction between irrigation regime and putrescine concentration significantly influenced cineole synthase (CS), sabinene synthase (SS), and bornyl diphosphate synthase (BPPS) relative expression. The highest concentration of 1,8-cineole, camphor, α-thujone, β-thujone, CS, SS, and BPPS were obtained in the irrigation regime of 80% ASWD with the application of 0.75 mM putrescine. There was high correlation between expression levels of the main monoterpenes synthase and the concentration of main monoterpenes. The observed correlation between the two enzyme activities of ascorbate peroxidase (APX) and catalase (CAT) strongly suggests they have coordinated action. On the other hand, the highest peroxidase (PO) and superoxide dismutase (SOD) concentrations were obtained with the application of 0.75 mM putrescine under the irrigation regime of 40% ASWD. Putrescine showed a significant increase in LAI and RWC under water deficit stress. There was an increasing trend in endogenous putrescine when putrescine concentration was increased in all irrigation regimes. Overall, the results suggest that putrescine may act directly as a stress-protecting compound and reduced H_2_O_2_ to moderate the capacity of the antioxidative system, maintain the membrane stability, and increase secondary metabolites under water deficit stress.

## Introduction

Water deficit stress is considered the most limiting environmental factor for the growth and yield of many plant species in arid and semi-arid regions. Water deficit stress affects plants at the morphological, physiological, biochemical, and molecular level^1^.One way to deal with water shortages is to cultivate herbs that have more tolerance. Generally, 49% of medicinal and aromatic plants whose native habitat is the Mediterranean region are notably tolerant and adaptable to dry conditions^2^. In addition to their economic value, these plants are proper crops for cultivation in dry areas^3^. There is a remarkable demand for sage (*Salvia officinalis* L.). and various related products all over the world. Accommodating this demand requires an increase in its cultivation area under water deficit conditions, particularly in arid and semi-arid areas. Sage was introduced in old Latin as a medicinal plant. The genus salvia is one of the best known genera due to monoterpenes as major compounds used in food and pharmaceutical industries^4^. Geranyl diphosphate (GPP) is the widely accepted common substrate for monoterpene biosynthesis. Three of the most important monoterpene synthases in sage are cineole synthase, sabinene synthase and bornyl diphosphate synthase. The cineole synthase (CS) produces in one step 1,8-cineole. The ( +)-sabinene synthase (SS) catalyzes the production of sabinene, which undergoes further rearrangements leading to the two major monoterpenes α- and β-thujone. The ( +)-bornyl diphosphate synthase (BPPS) produces bornyl diphosphate, which is subsequently hydrolyzed to borneol and then oxidized to camphor^5^. These compounds have potent antioxidative and anti-inflammatory effects^6^.

Zwenger and Basu^7^ reported that many factors, i.e. water deficit, nutrient, ozone, and mechanical stress, contribute to the up-adjustment of terpene synthesis genes in Arabidopsis. The essential oil yield and compounds in plants may change in water deficit stress. Plants generally produce higher levels of secondary metabolites under water deficit stress^8^. Increases in percentages of essential oils and main compounds have been reported in both *Ocimum basilicum* and *Ocimum americanum* under water deficit stress^9^. A study by Nowak et al.^10^ determined that the total content of monoterpenes in sage is significantly increased by moderate water deficit stress. The production of secondary metabolites, including glycyrrhizin, in liquorice plants and gene expression levels involved in their biosynthesis are strongly correlated to growth conditions^11^. It has been suggested that signal molecules may be employed directly or indirectly in the production of plant secondary metabolites^12^. The signal components involved in monoterpene biosynthesis might therefore serve as potential allelochemicals for the induction of monoterpenes. Researchers have found that the expression of monoterpene synthase increased with increasing concentrations of gibberellin but decreased when gibberellin biosynthesis was blocked with daminozide. Increasing concentrations of gibberellin also increased 1,8-cineole and camphor contents^13^.

Water deficit stress reduces plant growth by reducing cell turgor and relative water content (RWC), which in turn decreases cell elongation and development, consequently reducing leaf area^14^, and an imbalance between antioxidant defenses and the amount of reactive oxygen species (ROS) leads to oxidative stress under water deficit conditions^15^. Excessive accumulation of ROS can destabilize cell membranes and cause direct damage to DNA, pigments, proteins, lipids, and other essential cellular molecules, leading to cell death and loss of biomass^16^. Enhanced antioxidant enzyme activity helps in ROS scavenging and protecting against stress^16^. superoxide dismutase (SOD), peroxidase (PO), catalase (CAT), and Ascorbate peroxidase (APX) activity is responsible for quenching ROS. Indeed, they are usually activated when excessive ROS is generated to protect plants against oxidative damage under various types of stress^17^. As the first line of protection against ROS, SOD catalyzes the anion superoxide (O2−) to O2 and H2O2; then CAT catalyzes the decomposition of the H2O2 to water and molecular oxygen and PO works in the extra-cellular space for scavenging H2O2. PO expression was increased by the application of putrescine and PEG^18^. A survey of the literature indicated that salicylic acid (SA) can affect antioxidant enzyme activities and cause a moderate increase in the content of ROS such as hydrogen peroxide (H_2_O_2_)^19^, which acts as a second messenger in regulating plant defense responses.

Polyamines (PAs) are considered plant growth regulators with multiple functions and are implicated in many processes of plant growth and development, such as cell division and DNA replication. In plant cells, putrescine, spermidine and spermine are three major constituents of PAs^20^. Putrescine is produced from arginine or ornithine, while putrescine and S-adenosylmethionine are precursors for production of spermidine and spermine^21^. Overexpression of arginine decarboxylase and S-adenosylmethionine decarboxylase, the key genes for polyamine synthesis, resulted in elevated levels of putrescine, spermidine and spermine and increased water deficit stress tolerance in transgenic rice and tobacco plants^22^, while down regulation of S-adenosylmethionine decarboxylase gene declined spermidine and spermine levels and PAs and led to decreased water deficit stress tolerance in transgenic rice plants^23^.

A prevalent physiological response in various plants under abiotic stress is the accumulation of proline. Proline, as a component of stress signal transduction pathways, could be the nitrogen and carbon source needed for stress recovery and finally to help enhance stress tolerance^24^. Kotakis et al.^25^ announced that putrescine (as buffer and osmolite), inducing increase in proline content, which leads to maintenance of leaf water status under stress. Excessive expression of putrescine revealed both the up- and down-regulation of various stress-responsive, hormone and signaling-related genes, involved in the biosynthesis of auxin, ethylene, ABA, gibberellin and SA. Also, genes for auxin transport, genes coding for auxin-responsive proteins, ethylene and ABA-responsive transcriptional factors, and also jasmonate -induced proteins were also noticed, which confirm the dual role of putrescine and PAs in general: direct protection and participation in acclimation signaling pathways^26^. ROS derived from PAs oxidation is urgent in order to trigger stress response signaling. However, the size and rate of its accumulation determines cell fate, which means that ROS should not exceed specific thresholds; if so, it no longer signals the expression of stress genes but, instead, triggers programmed cell death^27^. Typically, when cellular PAs contents are up, their catabolism also augments, the levels of H_2_O_2_ increase, and various ROS as well as the anti-oxidative system (enzymatic and non-enzymatic) is also induced, hence their roles in preventing damage from stress are beneficial as well as deleterious. This is consistent with the notion that an augment in cellular PAs titers contributes to both sides of the ROS-anti-oxidative equation under stress^28^. Exogenous PAs help to improve the function and activation of antioxidant enzymes under water deficit stress.

In generally, secondary metabolites (such as isoprenoids, phenols or alkaloids) production can be directly enhanced by using deliberate water deficit stress. This enhancement can be reached by applying special irrigation regimes that are both simple and inexpensive, but this approach requires intense examination to optimize secondary metabolites (such as isoprenoids, phenols or alkaloids)^8^. In the current study, putrescine in different concentrations was employed as an elicitor to study the physiological, biochemical, and molecular changes in sage under water deficit stress. The main goal of this study was to determine the effects of water deficit stress and putrescine on the contents of three main monoterpenes in sage. It was hypothesized that the expression pattern of the genes involved in monoterpene biosynthesis pathways and their connection with increases in essential oil compounds under water deficit stress and putrescine may facilitate the production of economically valuable medicinal plants through genetic engineering techniques.

## Materials and methods

### Plant material, growth conditions, and sampling

The plants used are not wild and cuttings were prepared from one-year-old mother plants of *Salvia officinalis* L. in the medicinal farm of the Institute of Medicinal Plants & Natural Products Research, Iranian Academic Center for Education, Culture & Research (ACECR) (Karaj, Iran). A total of 48 sage plants were sown in 48 plastic pots with a diameter of 19 cm, height of 15.2 cm and volume of 3 L, containing a 1: 1: 1 mixture of (peatmoss: soil: sand). The physical and chemical properties of the field soil were assessed as follows: (Total N: 0.121%; T.N.V: 5.5%; organic carbon: 1.034%; field capacity (FC): 22.51%; permanent wilting point (PWP): 8.01%; E.C: 2.610 dSm^−1^, texture: Silt loam; pH: 7.51. Initial soil test values (mg kg^−1^) for P, K, Fe, Mn, Zn, Cu and B were 269, 68.8, 4.17, 8.91, 0.86, 1.09, and 1.77, respectively. Fertilization was not done. The growth conditions were 16/8 h (light/dark) and the light intensity was 300 μmol photons m^−2^ s^−1^ ^29,30^. Also, heating, cooling, relative humidity and vapor pressure deficit of growth chamber were set with 65 Ḟ, 75 Ḟ, %70 and 0.55 kPa, respectively. A factorial experiment was performed in a completely randomized design (CRD) with three replications. Treatments included irrigation regimes and putrescine, as follows:

1. Irrigation after depletion of 20% available soil water (control).
2. Irrigation after depletion of 40% available soil water.
3. Irrigation after depletion of 60% available soil water.
4. Irrigation after depletion of 80% available soil water.

Four concentrations of putrescine (distilled water (0), 0.75, 1.5, and 2.25 mM) were also applied. Foliar application of putrescine was performed one week before applying irrigation regimes. To investigate proper treatments, plants must have similar masses of foliage before treatments are applied. The plant height, number of nodes, number of leaves per plant and leaf area were 24 cm, 11, 52 and 8 cm^2^, respectively. Therefore, no water stress or putrescine were applied in the first 14 days of the growth cycle. During this period, all pots were irrigated when 20% of the available soil water was depleted (ASWD). The pots in unstressed control were irrigated daily and distance between upper and lower limits of water uptake was 20% available soil water which was determined by TDR. After leaf emergence, foliar application was conducted with 15 mL of putrescine for all aboveground parts of each plant. The depth of the irrigation water was assigned based on the available soil water and calculated using the following equations:

1 $${\text{ASWD}} = \left( {\theta {\text{FC}} - \theta {\text{PWP}}} \right) \times {\text{D}} \times {1}00$$

2 $${\text{Id}} = {\text{ASW}} \times \rho$$

3 $${\text{Ig}} = \left( {{\text{Id}} \times {1}00} \right)/{\text{Ea}}$$

where FC and PWP (%) are the volumetric soil water amounts, D (cm) is the soil layer depth, Id is the irrigation depth (cm), ρ is the fraction of ASW that can be depleted from the root zone (20%, 40%, 60%, and 80%), Ig is the coarse depth of irrigation (cm), and Ea is the irrigation efficiency (%) assumed at an average of 65%^31^. The applied irrigation water was measured (based on Eq. (3)) at each irrigation^32^. Irrigation treatments were implemented based on the maximum allowable depletion (MAD) from the percentage of ASWD. Each treatment was irrigated when the available soil water reached its threshold value^31^. The applied treatments were 20%, 40%, 60%, and 80% MAD of ASWD. A TDR probe (Time-Domain Reflectometry, Model TRIME-FM, Germany) was applied to measure soil water amount at a depth of 12.5 cm (root zone of sage). TDR probes were taken from the first experiment to the last experiment. Sampling was conducted from the sage leaves one week after applying the irrigation regimes. Also, samples from all treatments were collected on the same day in the morning. The expression rates were considerably higher in young leaves (first and second nodes) than in fully mature leaves (fifth and upper nodes)^30^. Accordingly, only first and second node leaves were used. These samples were cleaned with ethanol and paper tissues to remove any surface contamination, immediately frozen in liquid nitrogen, and stored at − 80 °C for use in measuring physiological attributes and RNA extraction.

### RWC, LAI (leaf area index) and putrescine endogenous

The length of time for the discs to reach saturation varies with species and conditions. Studies have shown that water uptake by leaf discs is initially rapid for several hours, followed by a slower rate uptake that can persist as long as the floated discs remain healthy. This usually occurs within 3 to 6 h. Our tests have shown that water uptake by leaf discs is initially rapid for four hours, followed by a slower rate uptake that could continue as 6 h as the floated discs remain healthy. On the other hand, errors arising from growth was minimized by growth inhibitors or by floating the discs at 4 °C. The water content of plants was easily determined by weighing the material immediately after sampling, drying at 75 °C and reweighing 24 h later.

To calculate RWC, 20 leaf discs (around 1 cm in diameter) were punched, and their fresh weight (FW) was recorded. The same leaf discs were kept in petri dishes containing distilled water for six hours to record saturation weight (SW), and after that discs were oven dried at 75 °C for 24 h to record the dry weight (DW). Calculations were performed using the formula: RWC = (FW – DW)/ (SW − DW)^33^.

Leaf tissue was ground under liquid nitrogen using a mortar and pestle. Putrescine extraction followed by HPLC quantification were carried out in accordance with Lütz et al.^34^.

$${\text{LAI}} = {\text{Sum}}\;{\text{of}}\;{\text{leaf}}\;{\text{area}}\left( {{\text{cm}}^{{2}} } \right)/{\text{Ground}}\;{\text{area}}\;{\text{where}}\;{\text{the}}\;{\text{leaves}}\;{\text{were}}\;{\text{collected}}\left( {{\text{cm}}^{{2}} } \right).$$

### RNA preparation and cDNA synthesis

Total RNAs were extracted from the sage leaves using a RNeasy Plant Mini Kit (QIAGEN GmbH, Hilden, Germany) according to the manufacturer’s instructions. RNA quality and concentrations were determined using 1.0% agarose gel electrophoresis and analysis by a NanoDrop 2000/2000c Spectrophotometer (Wilmington, DE 19,810 U.S.A.). Moreover, cDNA was synthesized with a SuperScript III Reverse Transcriptase reagent kit (Thermo Fisher Scientific, USA).

### Real*-*Time quantitative polymerase chain reaction (qRT-PCR)

qRT-PCR Primers for the assayed genes were designed according to the GenBank accession (Table 1). qRT-PCR was conducted with a Corbett Model (Rotor gene 6000). The qRT-PCR reaction for target gene transcript amplification was carried out in a final volume of 25 μL containing PCR buffer, 2.5 μL MgCl2, 0.5 μL dNTP, 2.5 μL of SYBR green I (Sigma-Aldrich), 0.1 μL of Taq, 1.25 μL of each forward and reverse primer, and 2 μL diluted cDNA. The PCR reaction conditions were denaturation at 95 °C for 5 min followed by 45 cycles at 95 °C for 20 s, primer annealing at 60 °C for 20 s, extension at 72 °C for 30 s, and a final extension at 72 °C for 2 min. Melting curve analysis include 95 °C for 60 s, 55 °C for 90 s, and 60 °C for 1 s. A housekeeping gene, glyceraldehyde-3-phosphate dehydrogenase C2 (GapC2), was used as the control to account for variations in cDNA template levels^35,36^. All reactions were done in triplicate.

**Table 1:** Description of reference genes and the monoterpene synthase primer sequences for qRT-PCR.

Gene	GeneBank accession number	Primer name	Primer sequence
1 ,8 cineole synthase	AF051899	CS (FW)	5́-TTCAAGCACAATTTCAACAAGAG-3ʹ
		CS (RV)	5́-AGCGTACCATAAATATCAAAGAC-3ʹ
Bornyl diphosphate synthase	AF051900	B-PP-S (FW)	5́-TATTTCACAGCTCTTGGATTCAG-3ʹ
		B-PP-S (RV)	5́-TGTAACATTCCCTTCGTATCTTG-3ʹ
Sabinene synthase	AF051901	SS (FW)	5́-AGGTGGTGATGAAATTGATGAAG-3ʹ
		SS (RV)	5́-ATATTGAAGTTGAGTTTGGCGAG-3ʹ
Glyceraldehyde-3-phosphate dehydrogenase C2	JN083806.1	Gap C2 (FW)	5́-CAGTGTATTGATGGATGGTATTC-3ʹ
		Gap C2 (RV)	5́-CCAAACTCACTTACTTCAAACAG-3ʹ

### Essential oil extraction

The shade-dried foliage of collected sage was extracted by hydro-distillation in a Clevenger device with double-distilled water. The obtained essential oil was separated from the aqueous layer using a 100 ml capacity separatory hopper (a piece of laboratory glassware used in liquid–liquid extractions). The collected surplus aqueous essential oil was dried over anhydrous sodium sulfate. After extraction, the essential oil was conserved in vials sealed at 4 °C until GC/MS evaluation.

### Identification of essential oil composition (GC–MS analysis)

Gas chromatography/mass spectrometry (GC/MS) analysis was accomplished using a Thermoquest-Finnigan TRACE GC–MS instrument (Manchester, UK) provided with the same gas chromatography situation as mentioned for the GC analyses. The percentages of compounds were calculated by the area normalization method on GC–MS chromatogram, without considering response factors. Helium was utilized as the vector gas at a flow rate of 1.1 ml min^−1^ with a split ratio of 1:50, and ionization voltage was 70 eV. The constituents of the essential oil were analyzed based on the retention index (RI) of the series of n-terpenes (C5h8)n and the oil on a Ph-5 column under the same chromatographic conditions. Single constituents were identified based on comparisons of their mass spectra fragmentation designs with standard ones found in the literature in wiley libraries or with those of valid compounds^37^.

### Glycine betaine (GB), proline and total reducing sugars (TRS)

GB was determined using the method of Grieve and Grattan^38^, in which 5 ml of toluene-water mixture (0.5% toluene) was added to 0.1 g dried ground material. Afterwards, tubes were shaken for 24 h at 25 °C, the extract was filtered, and the volume was made to equal 100 ml. Then, 1 ml of 2 N HCl solution was added to 1 ml of filtrate, and an aliquot of 0.5 ml from this solution was mixed with 0.1 ml of potassium tri-iodide solution. After placing the mixture in an ice bath for 90 min, 4 ml of 1,2 dichloroethane was added to it. The optical density was determined spectrophotometrically at 365 nm.

Proline was estimated using the method described by Bates et al.^39^.

To measure total reducing sugars, the method introduced by Dubois et al.^40^ was employed.

### Antioxidant enzymes (SOD, PO, APX and CAT) and 2,2-diphenyl-1-picrylhydrazyl (DPPH)

Leaf samples (500 mg) were pulverized in Na–P buffer (pH 7.0) containing 1 mM EDTA and 1% soluble polyvinyl pyrrolidone (PVP) using a mortar and pestle. The leaf extract was then centrifuged at 12,000 g for 20 min at 4 °C. The enzyme extracts obtained were used to determine the activity levels of SOD, CAT, PO, and APX enzymes. Enzyme activity was expressed as enzyme unit (EU) mg^−1^ protein. Furthermore, the extraction buffer for ascorbate peroxidase enzyme contained 1 mM ascorbic acid.

The protein concentration was analyzed using the method reported by Bradford^41^.

To estimate SOD activity (EC 1.15.1.1), the Bayer and Fridovich^42^ method was used. Briefly, 3 ml reaction mixture (containing 100 mM phosphate buffer with pH 7.8), 13 mM methionine, 75 µM NBT, 0.1 mM EDTA, 2 µM riboflavin, and 100 µL enzyme extract was incubated under a light intensity of 3600 lx for 15 min, and the reaction was stopped by switching off the light. The absorbance was read at 560 nm.

PO activity (EC 1.11.1.7) was determined utilizing the method introduced by Herzog and Fahimi^43^. The reaction mixture contained 0.15 M sodium phosphate-citrate buffer (pH 4.4), 50% (w/v) gelatin, 0.6% H_2_O_2_, and 5 μl enzyme extract. Absorption levels were read at 465 nm (extinction coefficient 2.47 mM^−1^ cm^−1^) for 3 min.

To estimate APX activity (EC 1.11.1.11), the Nakano and Asada^44^ method was employed. The reaction mixture contained 50 mM Na–P buffer with pH 7.0, 0.5 mM ascorbic acid, 0.1 mM EDTA-Na2, 0.12 mM H_2_O_2_, and 20 μl of enzyme extract. Absorbance was read at 290 nm.

CAT activity (EC.1.11.1.6) was determined using the method of Aebi^45^. Enzyme extract (100 µl) was added to 900 µl reaction solution (containing 50 mM sodium phosphate buffer with pH 7.0 mM H_2_O_2_), and absorbance was read at 240 nm.

The free radical scavenging activity of DPPH was determined spectrophotometrically as described by Hung et al.^46^. In brief, 2 mL of different extract solutions (16–500 g/ml) were mixed with 2 mL of DPPH solution. The mixture was allowed to stand for 30 min to achieve complete reaction at room temperature. Finally, the absorbance of samples was determined at 515 nm.

### H_2_O_2_ and lipid peroxidation (MDA)

To calculate leaf H_2_O_2_^47^, 100 mg fresh tissue was extracted with 5 mL trichloro acetic acid (0.1%) and then centrifuged at 10,000 rpm for 10 min. Finally, supernatant was mixed with potassium phosphate buffer (pH 7.0) and potassium iodide (KI), and absorbance was read at 390 nm. Fresh plant was ground into a fine powder in liquid nitrogen and homogenized with 0.1% (w/v) trichloroacetic acid (TCA). The homogenate was then centrifuged at 12,000 rpm. Aliquots of the supernatant were mixed with 0.5% 2-thiobarbituric acid (TBA) (prepared in 20% TCA). First, the mixture was heated to 95 °C and then cooled on ice. The mixture was further centrifuged at 12,000 rpm. The absorbance of the supernatant was read at 532 and 600 nm.

The MDA content was calculated using its absorption coefficient at 155 mM^−1^ cm^−1^, after the non-specific absorbance was reduced to 600 nm. Finally, MDA content was expressed as nmol g^-1^ of fresh weight^48^.

### Statistical analysis

Amplification data was analyzed using Rotor-Gene 6000 Series software (version 1.7). The threshold cycle (Ct) values of the triplicate PCRs were averaged and the relative quantification of the transcript levels was determined using the comparative Ct method ^49^. The Ct value of the calibrator (the sample with the highest Ct value) was subtracted from every other sample to produce the ∆∆Ct value, and 2^-∆∆Ct^ was taken as the relative expression level for each sample. The data was subjected to analysis of variance (ANOVA) using SAS 9.3 software (SAS Institute, Cary, NC, USA). Mean comparisons were evaluated using the LSD Test at 5% probability levels. Pearson’s correlation coefficients were determined using the CORR procedure.

## Results

### RWC, LAI and endogenous putrescine

Based on the results of analysis of variance, putrescine concentration, irrigation regime, and the two-way interaction between irrigation regime and putrescine concentration significantly influenced RWC, LAI and endogenous putrescine (Table 2).

**Table 2:** Analysis of variance (mean square) of relative water content (RWC), endogenous putrescine, cineole synthase (CS), sabinene synthase (SS), bornyl diphosphate synthase (BPPS), 1,8-cineole, α-thujone, β-thujone, camphor, glycine betaine (GB), proline, total reducing sugars (TRS), superoxide dismutase activity (SOD), peroxidase activity (PO), ascorbate peroxidase activity (APX), catalase activity (CAT), 2,2-diphenyl-1-picrylhydrazyl (DPPH), Hydrogen peroxide (H_2_O_2_), lipid peroxidation (MDA) and leaf area index (LAI) of *Salvia officinalis* influenced by irrigation regimes (I) and putrescine concentrations (P).

Sources of variation	I	P	I × P	Error
DF	3	3	9	32
RWC	21,259.03**	67,051.47**	23,922.83**	83.19
Endogenous P	10,521.54**	325,915.86**	8066.24**	184.94
CS	0.11**	0.24**	0.05**	0.0005
SS	0.21**	0.64**	0.21**	0.0007
BPPS	0.004**	0.006**	0.002**	0.0001
1,8-cineole	15.50**	1066.01**	5.06**	0.88
α-thujone	88.49**	435.27**	19.70**	0.36
β-thujone	150.71**	1025.30**	42.33**	0.48
camphor	63.12**	350.47**	18.57**	0.23
GB	0.21**	0.64**	0.21**	0.0007
Proline	15.50**	1066.01**	5.06**	0.88
TRS	0.004**	0.006**	0.002**	0.0001
SOD	63.12**	350.47**	18.57**	0.23
PO	88.49**	435.27**	19.70**	0.35
APX	102.90**	856.03**	32.37**	0.33
CAT	150.71**	1025.31**	42.33**	0.48
DPPH	21,259.03**	67,051.47**	23,922.83**	83.19
H_2_O_2_	10,521.54**	325,915.86**	8066.24**	184.94
MDA	0.11**	0.24**	0.05**	0.0005
LAI	102.90**	856.03**	32.37**	0.33

*ns*: non-significant.

*α ≤ 0.05.

**: α ≤ 0.01.

**Figure 1:**
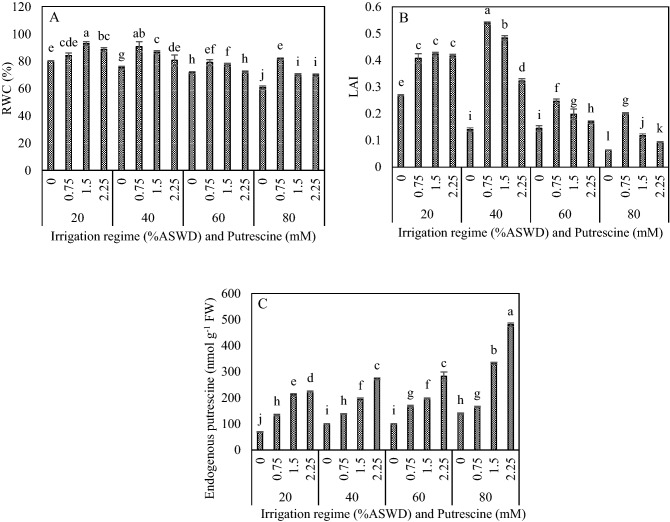
Interaction between irrigation regime and putrescine on RWC (**A**), LAI (**B**) and Endogenous putrescine (**C**). The different letters show significantly different at the level of 0.01. The error bars represent standard error.

The current results showed a decreasing trend in RWC with increasing water deficit stress. The highest RWC was obtained with the application of 0.75 and 1.5 mM putrescine under the irrigation regimes of 80% and 20% ASWD, respectively (Fig. 1A). Generally, the highest RWC (92.93%) was observed with the application of 1.5 mM putrescine under the irrigation regime of 20% ASWD (Fig. 1A). LAI was decreased under water deficit stress. The highest LAI was observed with the application of 0.75 mM putrescine under the irrigation regimes of 40%, 60%, and 80% ASWD (Fig. 1B). Generally, the highest LAI (0.54) was shown with the application of 0.75 mM putrescine under the irrigation regime of 40% ASWD (Fig. 1B). The current results revealed an increasing trend in endogenous putrescine when putrescine concentration was increased under irrigation regimes of 20%, 40%, 60%, and 80% ASWD. Moreover, the highest concentration of endogenous putrescine was obtained with the application of 2.25 mM putrescine under irrigation regimes of 20%, 40%, 60%, and 80% ASWD (Fig. 1C). Generally, the highest concentration of endogenous putrescine (480.00 nmol g^−1^ FW) resulted from the application of 2.25 mM putrescine under the irrigation regime of 80% ASWD (Fig. 1C).

### Gene Expression Assay

Expression analysis revealed that putrescine concentration, irrigation regime, and the two-way interaction between irrigation regime and putrescine concentration significantly influenced the relative expression levels of CS, SS, and BPPS (Table 2).

**Figure 2:**
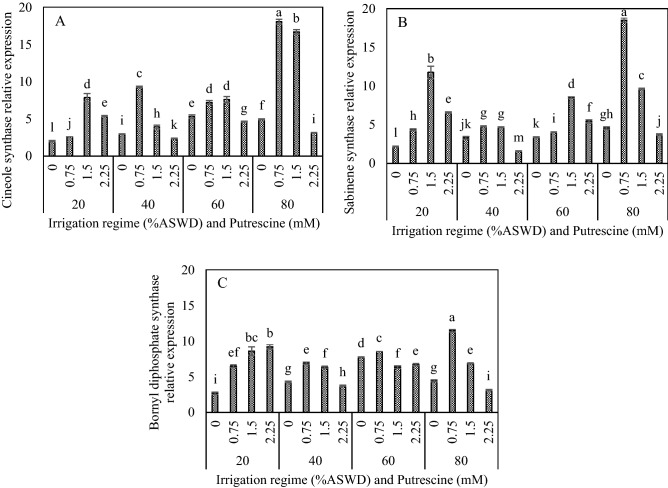
Interaction between irrigation regime and putrescine on cineole synthase (**A**), sabinene synthase (**B**) and bornyl diphosphate synthase (**C**) relative expression. The different letters show significantly different at the level of 0.01. The error bars represent standard error.

The CS, SS, and BPPS relative expression levels were increased under water deficit stress (Fig. 2A,B,C). The highest CS and SS relative expression levels were obtained with the application of 1.5 mM putrescine under the irrigation regime of 20% ASWD (Fig. 2A,B). The highest CS and BPPS relative expression level was achieved with the application of 0.75 mM putrescine under the irrigation regime of 40% ASWD (Fig. 2A,C). The highest SS and BPPS relative expression levels were obtained with the application of 1.5 and 0.75 mM putrescine under the irrigation regime of 60% ASWD, respectively (Fig. 2B,C). Generally, the highest relative expression levels of CS (18.06), SS (18.48), and BPPS (11.46) resulted from the application of 0.75 mM putrescine under the irrigation regime of 80% ASWD (Fig. 2A,B,C).

### Concentrations of 1, 8-cineole, α-thujone, β-thujone, and camphor

The results of GC/MS analyses revealed that the main compounds of the essential oils were α- thujone, β-thujone, camphor, and 1,8-cineole, respectively. These compounds accounted for more than 95% of the total monoterpenes amount. Therefore, the regulation of the biosynthesis of 1,8-cineole, camphor, α- thujone, and β-thujone was investigated in this study. The ANOVA results showed that putrescine concentration, irrigation regime, and the two-way interaction between irrigation regime and putrescine concentration significantly influenced the concentrations of 1,8-cineole, α-thujone, β-thujone, and camphor (Table 2).

**Figure 3:**
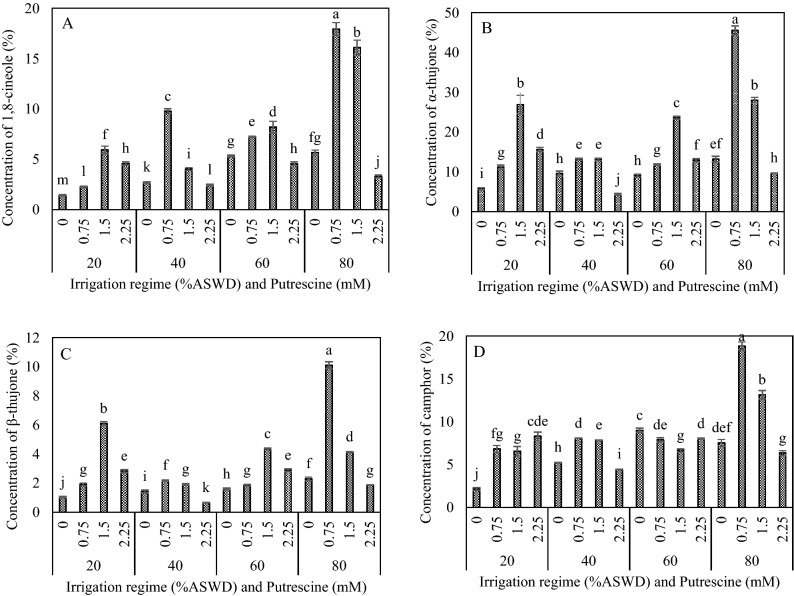
Interaction between irrigation regime and putrescine on concentration of 1,8-cineole (**A**), α-thujone (**B**), β-thujone (**C**) and camphor (**D**). The different letters show significantly different at the level of 0.01. The error bars represent standard error. (Retention Index = 1031, 1103, 1115 and 1144, respectively).

The concentrations of 1,8-cineole, α-thujone, β-thujone, and camphor were increased under water deficit stress (Fig. 3A,B,C,D). Also, the highest concentrations of 1,8-cineole, α-thujone, and β-thujone were obtained under irrigation regime of 80% compared to irrigation regime of 20% (Fig. 3A,B,C). The highest concentrations of 1,8-cineole, α-thujone, and β-thujone were obtained with the application of 1.5 mM putrescine under irrigation regimes of 20% and 60% ASWD (Fig. 3A,B,C). The highest concentration of camphor was observed with the application of 2.25 mM putrescine and distilled water under irrigation regimes of 20% and 60% ASWD, respectively (Fig. 3D). The highest concentrations of 1,8-cineole, β-thujone, and camphor were obtained with the application of 0.75 mM putrescine under irrigation regimes of 40% ASWD (Fig. 3A,C,D). Generally, the highest concentration of 1,8-cineole (17.95%), α-thujone (45.50%), β-thujone (10.10%), and camphor (18.83%) were observed in the irrigation regime of 80% ASWD with the application of 0.75 mM putrescine (Fig. 3A,B,C,D).

### Glycine betaine (GB), proline and total reducing sugars (TRS)

The analysis of variance results showed that putrescine concentration, irrigation regime, and the two-way interaction between irrigation regime and putrescine concentration significantly influenced significantly GB, proline, and TRS (Table 2).

**Figure 4:**
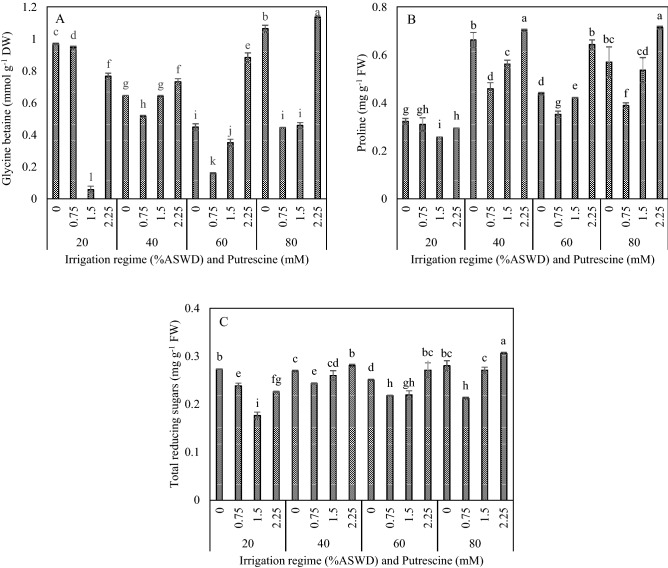
Interaction between irrigation regime and putrescine on glycine betaine (**A**), proline (**B**) and total reducing sugars (**C**). The different letters show significantly different at the level of 0.01. The error bars represent standard error.

The highest GB and proline contents were obtained under irrigation regime of 80% compared to irrigation regime of 20% (Fig. 4A,B). The lowest GB, proline and TRS contents were obtained with the application of 1.5 and 0.75 mM putrescine under irrigation regimes of 20% and 40% ASWD, respectively (Fig. 4A,B,C). Moreover, the lowest GB and proline content were observed with the application of 0.75 mM putrescine under irrigation regime of 60% ASWD (Fig. 4A,B). Generally, the lowest GB (0.06 mmol g^−1^ DW), proline (0.26 mg g^−1^ FW), and TRS (0.18 mg g^−1^ FW) contents were obtained with the application of 1.5 mM putrescine under the irrigation regime of 20% ASWD (Fig. 4A,B,C).

### Antioxidant enzymes (SOD, PO, APX, and CAT) and DPPH

Based on the results of the analysis of variance, putrescine concentration, irrigation regime, and the two-way interaction between irrigation regime and putrescine concentration significantly influenced antioxidant enzymes (SOD, PO, APX, and CAT) and DPPH levels (Table 2).

**Figure 5:**
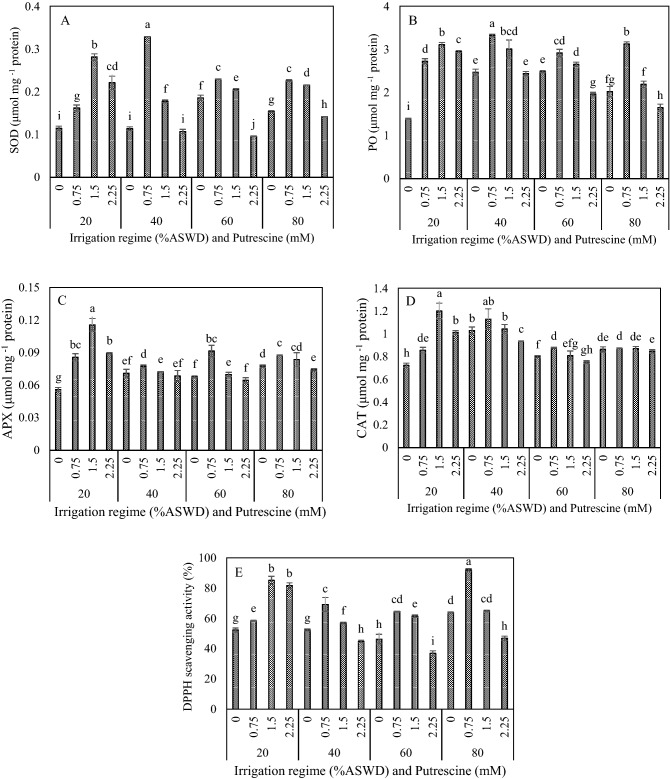
Interaction between irrigation regime and putrescine on SOD (**A**), PO (**B**), APX (**C**), CAT (**D**) and DPPH (**E**). The different letters show significantly different at the level of 0.01. The error bars represent standard error.

SOD, PO, APX, CAT, and DPPH values were increased under water deficit stress (Fig. 5A,B,C,D,E). The highest SOD, PO, and APX values were obtained with the application of 1.5, 0.75, and 0.75 mM putrescine under the irrigation regimes of 20%, 40%, and 60% ASWD, respectively (Fig. 5A,B,C). Moreover, the highest SOD and PO values were observed with the application of 0.75 mM putrescine under the irrigation regime of 80% ASWD (Fig. 5A,B). Furthermore, the highest CAT resulted from the application of 1.5 and 0.75 mM putrescine under the irrigation regimes of 20% and 60% ASWD, respectively (Fig. 5D). Generally, the highest SOD (0.33 μmol mg^−1^ protein) and PO (3.32 μmol mg^−1^ protein) values were obtained with the application of 0.75 mM putrescine under the irrigation regime of 40% ASWD (Fig. 5A,B), and the highest APX (0.11 μmol mg^−1^ protein) and CAT (1.20 μmol mg^−1^ protein) levels were obtained with the application of 1.5 mM putrescine under the irrigation regime of 20% ASWD (Fig. 5C,D). The highest DPPH was also obtained by applying 0.75 mM putrescine under the irrigation regimes of 40%, 60%, and 80% (Fig. 5E). Generally, the highest DPPH (91.89%) was observed with the application of 0.75 mM putrescine under the irrigation regime of 80% (Fig. 5E).

### H_2_O_2_ and MDA

The analysis of variance results showed that putrescine concentration, irrigation regime, and the two-way interaction between irrigation regime and putrescine concentration significantly influenced significantly H2O2 and MDA (Table 2).

**Figure 6:**
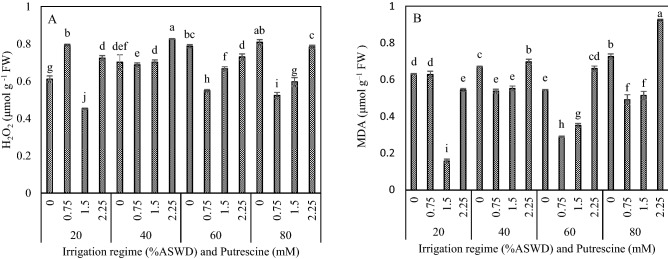
Interaction between irrigation regime and putrescine on H_2_O_2_ (**A**) and MDA (**B**). The different letters show significantly different at the level of 0.01. The error bars represent standard error.

The values of H_2_O_2_ and MDA increased under water deficit stress (Fig. 6A,B). The lowest H2O2 and MDA were obtained with the application of 1.5 and 0.75 mM putrescine under irrigation regimes of 20% and 60% ASWD, respectively (Fig. 6A,B). Moreover, the lowest H2O2 was obtained with the application of 0.75 mM putrescine under irrigation regime of 80% (Fig. 6A). Generally, the lowest H_2_O_2_ (0.45 μmol g^−1^ FW) and MDA (0.16 μmol g^–1^ FW) content were obtained with the application of 1.5 mM putrescine under the irrigation regime of 20% ASWD (Fig. 6A,B).

## Discussion

The current results showed a significant increase in compatible osmolytes (proline and GB) under the irrigation regime of 80% ASWD. Compatible osmolytes aid in the maintenance of turgor and stabilize macromolecular structures in response to stress^50^. The increase in sage proline in irrigation regimes of 40%, 60% and 80% indicates the important role of this osmolyte under water deficit stress. Indeed, the accumulation of proline and GB in stressed sage plants maintain membrane integrity, reduce oxidation of lipid membranes, stabilize ROS scavenging enzymes, and scavenge free radicals^51^. Generally, increases in compatible osmolytes stabilize redox potential and NAD(P) + /NAD(P)H ratio to prevent oxidative damage under stress conditions^52^.

In present study, LAI reduced under water deficit stress. On the other hand, this decrease was greater in irrigation regimes of 60% and 80% than 40% ASWD, which could be due to leaves with a smaller size and a higher falling rate. Exogenous application of 0.75 mM putrescine under irrigation regimes of 40%, 60% and 80% ASWD improved leaf area index. The highest LAI was obtained in irrigation regime of 40% ASWD under exogenous application of 0.75 mM putrescine. This improvement may be attributed to maintenance of LAI (assimilatory surface) due to high turgor and sustained photosynthetic ability of the crop by protecting the photosynthetic machinery from reactive oxygen species produced during drought stress and by increasing Rubp content under drought condition. Similarly, the positive effects of exogenously putrescine on LAI and tolerance to abiotic stresses in different plant species have been reported by others^53^.

In response to water deficit stress, RWC decreased in sage plants. Decreases in RWC subject the cell membranes to changes such as penetrability and decreased sustainability under water deficit stress^54^, that probably aims to create osmotic adjustment^55^. There was a negative correlation between proline and RWC (− 0.56; *P* < 0.05) (Table 3). Indeed, the decrease in RWC in irrigation regime of 80% AWSD were possibly due to the accumulation of proline and GB. Foliar application of putrescine alleviated the detrimental effects of water deficit stress and increased RWC considerably. These results are concordant with those of Farooq et al.^53^. The possible effect of foliar spray is related to the fact that while in direct contact with leaf surface, PAs improved the water status of epidermal cells and underlying cells^53^. It seems that the contribution of putrescine to osmotic adjustment can be considered as a mechanism to retain RWC for better growth and productivity. Two reasons for the responses of PAs under different adverse environmental conditions might be the ability to scavenge ROS and adjust osmosis^53^.

**Table 3:** Pearson’s correlation coefficients among relative water content (RWC), endogenous putrescine, cineole synthase (CS), sabinene synthase (SS), bornyl diphosphate synthase (BPPS), 1,8-cineole, α-thujone, β-thujone, camphor, glycine betaine (GB), proline, total reducing sugars (TRS), superoxide dismutase activity (SOD), peroxidase activity (PO), ascorbate peroxidase activity (APX), catalase activity (CAT), 2,2-diphenyl-1-picrylhydrazyl (DPPH), Hydrogen peroxide (H_2_O_2_), lipid peroxidation (MDA) and leaf area index (LAI) of *Salvia officinalis* influenced by irrigation regimes and putrescine concentrations.

	RWC	Endogenous putrescine	CS	SS	BPPS	1,8-Cineole	α-thujone	β-thujone	Camphor	GB	Prolin	TRS	SOD	PO	APX	CAT	DPPH	H2O2	MDA	LAI
RWC	1																			
Endogenous putrescine	− 0.22^ ns^	1																		
CS	0.04^ ns^	0.10^ ns^	1																	
SS	0.22^ ns^	0.07^ ns^	0.83**	1																
BPPS	0.41^ ns^	− 0.14^ ns^	0.66**	0.73**	1															
1,8-Cineole	− 0.06^ ns^	0.14^ ns^	0.98**	0.74**	0.58*	1														
α-thujone	0.15^ ns^	0.08^ ns^	0.88**	0.99**	0.71**	0.81**	1													
β-thujone	0.19^ ns^	0.05^ ns^	0.80**	0.99**	0.71**	0.70**	0.97**	1												
Camphor	− 0.05^ ns^	0.11^ ns^	0.88**	0.80**	0.75**	0.85**	0.83**	0.78**	1											
GB	− 0.42^ ns^	0.20^ ns^	− 0.52*	− 0.47^ ns^	− 0.64**	− 0.48^ ns^	− 0.48^ ns^	− 0.45^ ns^	− 0.32^ ns^	1										
Prolin	− 0.56*	0.49 ns	− 0.22^ ns^	− 0.36^ ns^	− 0.56*	− 0.13^ ns^	− 0.32	− 0.34^ ns^	− 0.12^ ns^	0.43^ ns^	1									
TRS	− 0.64**	0.35^ ns^	− 0.39^ ns^	− 0.60*	− 0.78**	− 0.28^ ns^	− 0.56*	− 0.59*	− 0.29^ ns^	0.77**	0.79**	1								
SOD	0.54*	− 0.11^ ns^	0.58*	0.48^ ns^	0.64**	0.55*	0.49^ ns^	0.44^ ns^	0.40^ ns^	− 0.66**	− 0.56*	− 0.69**	1							
PO	0.70**	− 0.28^ ns^	0.37^ ns^	0.43^ ns^	0.74**	0.31^ ns^	0.42^ ns^	0.40^ ns^	0.39^ ns^	− 0.67**	− 0.42^ ns^	− 0.75**	0.74**	1						
APX	0.45^ ns^	0.09^ ns^	0.40^ ns^	0.56*	0.63**	0.32^ ns^	0.52*	0.52*	0.34^ ns^	− 0.55*	− 0.52*	− 0.73**	0.65**	0.60*	1					
CAT	0.65**	− 0.05^ ns^	0.08^ ns^	0.17^ ns^	0.25^ ns^	0.03^ ns^	0.13^ ns^	0.13^ ns^	− 0.01^ ns^	0.42^ ns^	− 0.13^ ns^	− 0.42^ ns^	0.59*	0.69**	0.60**	1				
DPPH	0.49^ ns^	− 0.17^ ns^	0.64**	0.77**	0.71**	0.54*	0.75**	0.74**	0.55*	− 0.49^ ns^	− 0.64**	− 0.74**	0.74**	0.64**	0.76**	0.50*	1			
H_2_O_2_	− 0.12^ ns^	0.04^ ns^	− 0.70**	− 0.73**	− 0.69**	− 0.65**	− 0.74**	− 0.72**	− 0.58*	0.71**	0.43^ ns^	0.68**	− 0.66**	− 0.48^ ns^	− 0.74**	− 0.31^ ns^	− 0.72**	1		
MDA	− 0.52*	0.31^ ns^	− 0.42^ ns^	− 0.49^ ns^	− 0.65**	− 0.36^ ns^	− 0.48^ ns^	− 0.46^ ns^	− 0.20^ ns^	0.91**	0.69**	0.90**	− 0.66**	− 0.63**	− 0.63**	− 0.37^ ns^	− 0.58*	0.74**	1	
LAI	0.92**	− 0.23^ ns^	− 0.12^ ns^	− 0.02^ ns^	0.25^ ns^	− 0.17^ ns^	− 0.07^ ns^	− 0.05^ ns^	− 0.16^ ns^	− 0.23^ ns^	− 0.44^ ns^	− 0.44^ ns^	0.49^ ns^	0.65**	0.31^ ns^	0.64**	0.33^ ns^	0.01^ ns^	− 0.35^ ns^	1

*ns* non-significant.

*α ≤ 0.05.

**α ≤ 0.01.

In the present study, water deficit stress increased the production of H2O2 and MDA in plants, which is an indication of tissue damage, thus enhancing SOD, CAT, PO, APX and DPPH activities under water deficit conditions. The effectiveness of the antioxidant defense system function depends on the intensity of the water deficit stress^56^. The highest APX and DPPH concentrations for scavenging H2O2 and MDA protecting biomolecules were obtained in the 80% ASWD irrigation regime. This higher enzyme activity did not provide enough protection against ROS (H2O2) and MDA. Indeed, to improve the damage oxidative stress imposes on sage, foliar applications of putrescine (0.75 and 1.5 mM) are required. Similar results were reported by Tajti et al.^57^ achieved similar results on cadmium stress in wheat by increasing putrescine levels. Negative correlations between SOD, PO, and APX and MDA were observed (− 0.66, − 0.63, and − 0.63; *P* < 0.01, respectively), GB (− 0.66, − 0.67, and − 0.55; *P* < 0.05 and 0.01, respectively), and TRS (− 0.69, − 0.75, and − 0.73; *P* < 0.01, respectively) (Table 3). There were also negative correlations between SOD and APX and proline (− 0.56 and − 0.52; *P* < 0.05, respectively) (Table 3). Also, there was high correlations between H2O2 and MDA (0.74; *P* < 0.01) (Table 3). The lowest compatible osmolytes and H2O2 and MDA and highest antioxidant enzymes including CAT and APX were obtained with the application of 1.5 mM putrescine; this showed the role of putrescine in decreasing of lipid peroxidation, stabilizing the cell membranes, preventing degrading of cell membranes by free radicals like H2O2. This response indicates a good H2O2 scavenging ability in the application of putrescine. Indeed, putrescine inhibits NADPH oxidase enzymes in cell walls, which ultimately leads to less H2O2 production in putrescine-treated plants^58^.

The observed correlation between the two enzyme activities strongly suggests coordinated action between APX and CAT. Yiu et al.^59^ reported similar results. In the present study, with the application of 0.75 mM putrescine, SOD and PO played an important role in protecting H2O2 under the 40% ASWD irrigation regime. On the other hand, SOD (as the first line of defense against ROS) and APX have significant negative correlations with H_2_O_2_ (− 0.66, − 0.74; *P* < 0.01, respectively) (Table 3). Indeed, probably putrescine substantially improved the impacts of water deficit stress on the membrane stability index in sage plants by binding to the negatively-charged phospholipid head group^60^. It is well documented that PAs (e.g., putrescine) are able to induce adaptive changes to maintain plasma membrane integrity under water deficit stress. Moreover, the enzymatic antioxidant activity enhanced by putrescine seems to be the result of de novo synthesis and/or the activation of the enzymes, mediated by the transcription and/or translation of specific genes^61^, that potentially aids stressed plants to resist against oxidative stress induced by water deficit stress. There were also high correlations between LAI, SOD, PO and CAT and RWC (0.92, 0.54, 0.70 and 0.65; *P* < 0.01, respectively) (Table 3). Moreover, there were positive correlations between PO and CAT and LAI (0.65 and 0.64; *P* < 0.01) (Table 3). So, the highest RWC, LAI, PO, CAT and SOD were obtained with the application of 0.75 mM putrescine and the 40% ASWD irrigation regime. Also, the highest RWC and CAT were obtained with the application of 1.5 mM putrescine and the 20% ASWD irrigation regime.

Elevation of ambient CO_2_ concentration results in a massive enhancement of photosynthesis, quite often the rate is doubled or even tripled. The absolute demands for ATP and NAD(P)H by the plant cell as well as the demand for ATP relative to NAD(P)H vary in response to growth, development and stress conditions^62^. A dominant concept is that water deficit stress, similar some other stresses, favors electron flux to O_2_ based on the idea that the regeneration of NADP^+^ cannot keep pace with NADPH generation. However, any significantly increased in the NADPH: NADP^+^ ratio will have deep effects on the regulation of photosynthesis due to the vital coupling of electron transport to ATP synthesis^63^. An important point (In terms of the sustainability of alternative electron flow) is the mean turnover times of ATP and NADPH in the chloroplast stroma, which do not trespass a few seconds at moderate to high rates of steady-state photosynthesis^64^. In addition to any raise of the Mehler reaction, over reduce of the stroma will favor engagement of other ATP generating processes such as cyclic electron transport and chlororespiratory pathways. Classical energy dissipating mechanisms for eliminating the overflow of electrons comprise non-photochemical quenching, photorespiration, and the xanthophyll cycle^65^. As a result, photosynthetic control over plastoquinol oxidation at the cytochrome b6f. complex will limit overreduced of PSI and join to build electron pressure in PSII^66^. Increased photosynthetic control will not only promote energy dissipation in PSII but will also tend to increase the likelihood of singlet oxygen production. Furthermore, the synthesis of secondary metabolites and biosynthesis of highly reduced compounds like isoprene are significantly involved in the dissipation of excess photosynthetic energy^67^. The amount of energy decomposed by isoprene emission could account for up to 25% of the net photosynthesis energy store under stress conditions^68^. Indeed, synthesis and increased secondary metabolites could be considered as the machinery to minimize the reduction equivalents.

The current results showed that monoterpene concentration, monoterpene synthesis, and H2O2 were increased under water deficit stress conditions with the highest contents obtained with the 80% ASWD irrigation regime. Indeed, it is well known that ROS as signal components are involved in the activation of monoterpene biosynthetic enzymes, and to some extent, oxidative bursts could induce monoterpene biosynthesis^69^. The highest levels of expression of the main monoterpene synthase and concentrations of main monoterpenes were obtained with the application of 0.75 mM putrescine and the 80% ASWD irrigation regime. On the other hand, there were positive correlations between CS, BPPS, and SS and 1,8-cineol, camphor, α-thujone, and β-thujone (0.98, 0.75 and 0.99/0.99; *P* < 0.01, respectively) (Table 3). The increase in the secondary metabolite contents in sage are due to a “passive” shift of biosynthesis as a result of an over-reduced status and an “active” up-regulation of the enzymes involved in the corresponding biosynthesis^30^.

The biosynthesis of terpenes is composed of two distinct paths: methylerythritol 4-phosphate (MEP) and mevalonate (MVA), occurring in the plastid and cytoplasm of plants, respectively. The MEP pathway is for the synthesis of carotenoids, isoprene, mono- and diterpenes, plant hormones [abscisic acid (ABA), gibberellins (GA)], phytol, the side chain of chlorophyll, tocopherols, phylloquinone, plastoquinones, etc^70^. Monoterpenes biosynthesis is located in plastids^71^. PAs in plants are not only found in cytoplasm, but also in specified organelles like mitochondria, chloroplasts, and vacuoles^72^. Our results showed the ratio of (1,8-cineol and CS), (camphor and BPPS) and (α- thujone, β-thujone and SS) were obtained (3.16 and 3.65 times), (2.50 and 2.59 times), and (3.43, 4.41, and 4.04 times) with the application of 0.75 mM putrescine compared to the application of distilled water under the irrigation regime of 80% ASWD. So, in explaining this result, it should be said that putrescine enters the leaves by penetrating the cuticle or through the stomata before entering the plant cell, where they can be practical in metabolism and are further transported to other parts through plasmodesmata. Therefore, putrescine and monoterpenes were probably produced in the MEP pathway (plastid) and essential oil content affected by putrescine. Schmiderer et al.^13^ achieved similar results; they reported that the expression of monoterpene synthase and contents of 1,8-cineole and camphor were increased with the application of gibberellins. Methyl jasmonate and SA have also been applied as abiotic elicitors to induce secondary metabolite biosynthesis as terpene metabolism in *Tanacetum parthenium*^73^.

The highest amount of DPPH was observed with the application of 0.75 mM putrescine under the irrigation regime of 80% ASWD. High correlations were observed between DPPH and CS, BPPS, SS, 1,8-cineol, camphor, α- thujone, and β-thujone (0.64, 0.71, 0.77, 0.54, 0.55, 0.75 and 0.74; *P* < 0.05, 0.01, respectively) (Table 3). There were also high correlations between SOD and CS, BPPS, and 1,8-cineol (0.58, 0.64, and 0.55; *P* < 0.05, 0.01, respectively), between PO and BPPS (0.74; *P* < 0.01), and between APX and BPPS, SS, α-thujone, and β-thujone (0.63, 0,56, 0.52, and 0.52; *P* < 0.05, 0.01, respectively) (Table 3). High correlations were also observed between DPPH and SOD, PO, CAT, and APX (0.74, 0.64, 0.50, and 0.76, respectively) (Table 3).

According to the current results, the content of compatible osmolytes and concentration of antioxidant enzymes were increased and decreased, respectively, with the application of 2.25 mM putrescine. There is evidence that SA causes a rise in the quantity of ROS in the cell, suggesting the existence of a self-induced SA H2O2 cycle^74^. This is not surprising, as a close correlation was recently reported between the endogenous PAs and SA contents^75^, which may be responsible for the negative effects of greater concentrations of putrescine. Some reports on SA have been in agreement with the current results^76^. The treatment of 2.25 mM putrescine resulted in an inhibitory effect compared with the 0.75 mM putrescine treatment. This is why stress-induced H2O2 accumulation was lower in the 0.75 mM than the 2.25 mM putrescine-treated plants. Putrescine reduced the accumulation of total monoterpenes in concentrations of 2.25 mM which, to some extent, can be attributed to excessive oxidative burst-induced putrescine in high concentrations. Indeed, there was a decreasing trend in the concentration of monoterpenes and the expression of monoterpene synthases genes with increasing putrescine concentrations under the irrigation regime of 80% ASWD; however, further studies are needed to determine whether the application of putrescine in high concentrations at the cellular level decrease monoterpenes in sage under water deficit stress conditions.

## Conclusion

The current results showed a foliar application of putrescine alleviated the detrimental effects of water deficit stress and considerably increased LAI and RWC. The lowest compatible osmolyte and H_2_O_2_ contents and the highest antioxidant enzymes including CAT and APX were obtained with the application of 1.5 mM putrescine, which showed the role of putrescine in decreasing of lipid peroxidation, stabilizing cell membranes and preventing the degradation of cell membranes by free radicals like H_2_O_2_. This response indicates a good H_2_O_2_ scavenging ability with the application of putrescine. In the present study, SOD and PO in the application of 0.75 mM putrescine played important roles in decreasing H_2_O_2_ under the irrigation regime of 40% ASWD. The highest levels of expression of the main monoterpenes synthase, concentrations of main monoterpenes, and DPPH were obtained with the application of 0.75 mM putrescine and the irrigation regime of 80% ASWD. PAs and monoterpenes were probably produced in the (MEP) pathway, and the essential oil content affected by putrescine. In general, the adequate putrescine concentration to trigger a best response is 0.75 mM. Indeed, putrescine could be a useful strategy to increase the main monoterpenes in sage plants. However, further researches are needed to determine the appropriate concentrations of exogenous putrescine under water deficit stress conditions in sage.

### Statements on plant material

The plant material in this manuscript complies with relevant institutional, national and international guidelines and laws and can be obtained from various respective centers worldwide. The plants used are not wild and were purchased from Institute of Medicinal Plants & Natural Products Research, Iranian Academic Center for Education, Culture & Research (ACECR) (Karaj, Iran).
